# Healthcare Professionals’ Knowledge and Beliefs on Antibiotic Prophylaxis in Cesarean Section: A Mixed-Methods Study in Benin

**DOI:** 10.3390/antibiotics11070872

**Published:** 2022-06-28

**Authors:** Angèle Modupè Dohou, Valentina Oana Buda, Severin Anagonou, Françoise Van Bambeke, Thierry Van Hees, Francis Moïse Dossou, Olivia Dalleur

**Affiliations:** 1 Louvain Drug Research Institute, Université Catholique de Louvain, Avenue Emmanuel Mounier 73, 1200 Brussels, Belgium; francoise.vanbambeke@uclouvain.be (F.V.B.); olivia.dalleur@uclouvain.be (O.D.); 2 Faculté des Sciences de la Santé, Université d’Abomey Calavi, Cotonou 01 BP 188, Benin; anagonou_severin@yahoo.fr (S.A.); dosfm@yahoo.fr (F.M.D.); 3 Faculty of Pharmacy, “Victor Babes” University of Medicine and Pharmacy, Eftimie Murgu Square, No. 2, 300041 Timisoara, Romania; buda.valentina@umft.ro; 4 Center for Interdisciplinary Research on Medicines, Université de Liège, Place du 20 Août 7, 4000 Liège, Belgium; thierry_vanhees@outlook.com; 5 Service de Pharmacie Clinique, Cliniques Universitaires Saint-Luc, Avenue Hippocrate 10, 1200 Brussels, Belgium

**Keywords:** cesarean section, antibiotic prophylaxis practices, healthcare professionals, knowledge, beliefs, Benin

## Abstract

A low adherence to recommendations on antibiotic prophylaxis has been reported worldwide. Since 2009, cesarean sections have been performed under user fee exemption in Benin with a free kit containing the required supplies and antibiotics for prophylaxis. Despite the kit, the level of antibiotic prophylaxis achievement remains low. We conducted a convergent parallel design study in 2017 using a self-administered questionnaire and interviews to assess the knowledge and explore the beliefs of healthcare professionals regarding antibiotic prophylaxis in three hospitals. Of the 35 participants, 33 filled out the questionnaire. Based on the five conventional criteria of antibiotic prophylaxis, the mean level of knowledge was 3.3 out of 5, and only 15.2% scored 5 out of 5. From the verbatim of 19 interviewees, determinants such as suboptimal patient status health, low confidence in antibiotics, some disagreement with the policy, inappropriate infrastructures and limited financial resources in hospitals, poor management of the policy in the central level, and patient refusal to buy antibiotics can explain poor practices. Because of the dysfunction at these levels, the patient becomes the major determinant of adequate antibiotic prophylaxis. Policymakers have to consider these determinants for improving antibiotic prophylaxis in a way that ensures patient safety and reduces the incidence of antimicrobial resistance.

## 1. Introduction

In 2021, new research from the World Health Organization reported that the cesarean section (CS) rate was more than one in five (21%) of all childbirths globally. In developing countries, a rate of 8% of women gave birth by CS, with 5% in sub-Saharan Africa [1]. A cesarean section can be a life-saving procedure, and prevents poor obstetric outcomes [2]. CS decreases the rate of maternal and neonatal morbidity, with a positive impact on women, especially in developing countries, where the CS rate is in the range of 5–10% [3]. However, a number of CSs have been reported to be non-medically justified, and could be harmful to the mother and her baby [4].

In the absence of universal health insurance coverage, the fear of medical, social, and financial implications of childbirth by CS decreases its affordability in developing countries [5]. To reach the Millennium Development Goal N.3, which consists of achieving universal health coverage [6], several African countries have implemented a user fee exemption policy [7]. User fees contribute to the unaffordable cost burdens imposed on poor households, and represent one facet of the social exclusion experienced by these households [8]. In recent years, several countries in sub-Saharan Africa have introduced user fee exemption policies to facilitate access to various maternal health services, including cesarean section [8]. In April 2009, the Benin government set up a national agency and approved 48 care facilities for their CS user fees exemption policy [9]. This policy improved the affordability of CS and resulted in an increase in its rate from 3.7% to 6.4% three years later [10], and a rate of 5.1% in 2018, reported by UNICEF [11]. The WHO reported a decrease in maternal mortality from 471 in 2009 to 397 per 100,000 deliveries in 2017 [12]. Although studies have reported a link between CS and maternal mortality [3], the report “Femhealth” of Centre de Recherche en Reproduction Humaine et en Démographie-Benin in 2014 concluded with a mixed appreciation [10]. The agency funds, by retroactive reimbursement, a fixed sum of 100.000 FCFA “Francs de la Communauté Financière Africaine” (an average of US$167 or 153.40 euros) for each CS performed in the approved hospitals [13]. This sum covers the check-up costs before the medical intervention, a kit (containing the materials and drugs required, including injectable antibiotics: ampicillin, gentamicin, and metronidazole; and oral antibiotics: amoxicillin and metronidazole), surgery, blood transfusion if needed and hospitalization for seven days [9]. Several studies have evaluated different components of the user fee exemption policy and their impact on its implementation and patients’ opinions on CSs in Benin hospitals [14,15]. After our observational study performed in 2016 in four hospitals in Benin, based on the antibiotics in the kit, our data showed improper practices of antibiotic prophylaxis [16] regarding the five conventional criteria, namely, indication, choice of the molecule, the timing of administration, the dose administered, and the duration of administration [17]. Infections during pregnancy are common, and other conditions, such as malnutrition, obesity, anemia, bacterial vaginosis, diabetes, group B streptococcus infections, and CS, may increase the risk [18]. However, when a CS is medically justified, antibiotic prophylaxis is essential to prevent poor post-operative outcomes [19]. However, the use of antibiotics in pregnancy, including the CS section, has to be weighted regarding the risk for women and babies (lactation), and the existing threat of antimicrobial resistance [20,21]. In low-income countries such as Benin, because of the suboptimal health status and the lack of hygiene of the patients [22], and the unclean state of the hospitals and infrastructures [23], women are more at risk for infections. Post-CS infections induce a high rate of expenses for patients, since these infections’ treatment is not considered in the user fees exemption policies. From the data published in a study performed in Mali, 62.5–100% of women were infected post-partum, and expenses for antibiotics were higher than those of other post-partum complications [24]. Considering the burden infections represent for patients in low-income countries, prevention should be well-achieved. This study aimed to understand the determinants of the poor achievement of antibiotic prophylaxis by assessing and exploring healthcare professionals’ (HcPs) knowledge and beliefs on antibiotic prophylaxis. By providing relevant data on those determinants, our study will help policymakers to set up interventions for improving antibiotic prophylaxis in Benin, and other countries with the same challenges.

## 2. Results

### 2.1. Assessment of the Level of Knowledge of Antibiotic Prophylaxis

A total of 35 healthcare professionals agreed to take part in the survey. The assessment of the HcPs’ knowledge was based on data from 33 participants who completed the self-administered questionnaire. The response rate was 94.3% (33/35). Males represented 51.5% (17/33) of all participants; 60.6% (20/33) were nurse anesthetists; and the others (39.4%; 13/33) were physicians (anesthetists, anesthetists in specialization, and obstetricians). The median age and professional seniority were, respectively, 39.6 years old (range 23–57 years old) and 7.8 years (range = 1–22 years). The respondents’ demographic characteristics are displayed in Table 1.

**Figure 1:**
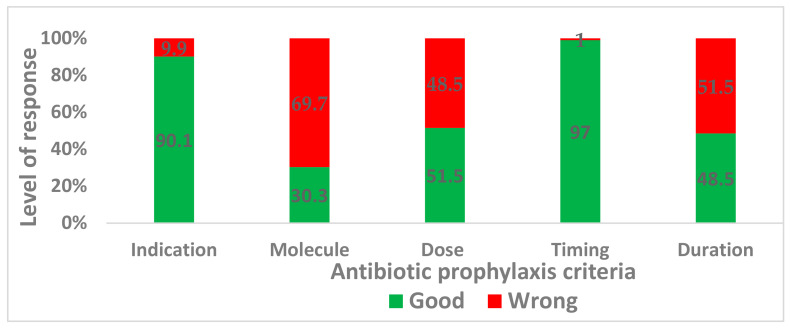
Level of response of the healthcare professionals.

From the data collected in the three hospitals, the indication and the timing of the antibiotic prophylaxis were the criteria that had the best scores: 90.1% and 97.0%, respectively. On the contrary, 69.7%, 48.5%, and 51.5% of the HcPs did not provide good responses to the choice of molecule, dose, or duration of administration of antibiotic prophylaxis, respectively. Only 5 medical doctors out of the 33 participants (15.2%) provided a good response to the five conventional criteria (scored 5/5). The mean level of knowledge was 3.3 out of 5. A total of 25 HcPs (75.8%) scored at least three out of five. The HcPs with certain seniority (more than 10 years) indicated the antibiotics of the kit as those recommended. The younger HcPs often indicated broad-spectrum antibiotics. The participants stated that they had not attended training on antibiotic prophylaxis since they started working, and all of them declared that antibiotic prophylaxis practice has to be improved in their hospital. The levels of the response of the HcPs on the five conventional criteria for antibiotic prophylaxis are represented in Figure 1.

For the three less-known criteria, we calculated the scores of the responses for each socio-professional category, with the results displayed in Table 2. From the analysis of the results, we noticed that the medical doctors (MDs) provided better responses than the nurses (score out of 5/5: 15.2% of MDs versus 0% of nurses; good dose: 69.2% of MDs versus 40% of nurses; good duration: 76.9% of MDs versus 30% of nurses), and 40% of the nurses did not know the antibiotic recommended for prophylaxis.

### 2.2. Interviews

The interviews were conducted over two months and included 19 healthcare professionals (9 doctors and 10 nurses; 11 women and 8 men) from the 33 respondents of the previous sample within the three hospitals. The study population ranged from 23 to 57 years in age. Ten face-to-face interviews and two focus groups of three and five participants, composed exclusively of nurse anesthetists, were conducted. All participating medical doctors preferred face-to-face interviews (not focus groups). The discussions lasted approximately 20–45 min for the individual interviews, and 36–50 min for the focus groups. Apart from the field notes of one of the interviewees, no field notes were taken for the recorded interviews. The obstetricians in one of the three hospitals did not give us the opportunity to conduct interviews with them. Data collection was stopped when we did not have other volunteer participants in the study period.

**Figure 2:**
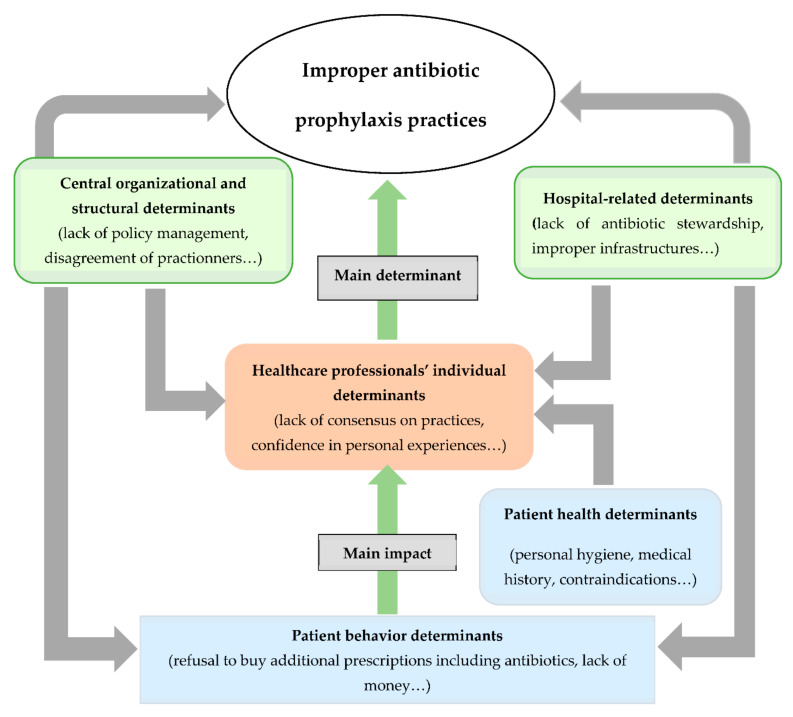
Organization of the five determinants of improper antibiotic prophylaxis practices.

Within the data emerging from the verbatim retranscription, five main determinants subdivided into 17 codes were inductively identified to influence antibiotic prophylaxis achievement in hospitals: patient health determinants, hospital-related determinants, healthcare professionals’ individual determinants, central organizational and structural determinants (policy management), and patient behavior determinants Figure 2. shows the organization of the five determinants of improper antibiotic prophylaxis practices.

#### 2.2.1. Patient Health Determinants

In the opinion of the HcPs, patients’ health determinants may influence antibiotic prophylaxis practices in terms of their personal hygiene, medical history, and occurrence of complications after a cesarean section. They also thought that the fact that some patients may already be contaminated with resistant germs has to be considered.

When we asked about the reasons that can lead to the choice of antibiotic for prophylaxis, one of the obstetricians, referring to patients’ hygiene and other required hygiene conditions, answered as follows:

*“But for me, it is above all the patients who do not wash themselves properly- the hygiene of the skin on which we are going to work on. The absence of shower before patients enter in the operating room. The conditions that must be met before the patients enters the operating room are not met.”*

(Gynecologist Medical Doctor 3, Hospital 2)

On the contrary, a nurse anesthetist argued that patient contraindications (allergies, renal failure, etc.) to the antibiotics in the kit (ampicillin and gentamicin) and the type of CS (emergency vs. planned) can impact HcPs’ attitudes toward antibiotic prophylaxis practices.

*“Now, there are some women for whom we do not use gentamicin. But if the woman is not allergic to ampicillin, we will use both.”*

(Nurse Anesthetist 4, Hospital 1)

*“We systematically administer 2 g of ampicillin and 160 mg of gentamicin if the woman has no history of hypertension.”*

(Nurse Anesthetist 2, Hospital 3)

In sum, it appears that the perception of safety is key to the choice of antibiotic prophylaxis in terms of the molecule, dose, and duration.

#### 2.2.2. Hospital-Related Determinants

Organization and conditions of care in the hospitals were frequently described as influencing antibiotic prophylaxis practices. Inadequate or inconstant hospital infrastructures with potential consequences for hygiene could influence the achievement of the antibiotic prophylaxis criteria. Then, in the Benin work context, the lack of important tools or an aseptic environment and improper operating rooms were mentioned as reasons for using different antibiotic prophylaxis practices than those recommended. Again, the perceived infectious risk related to contamination of the environment and the global safety of the patient drove the HcPs’ behavior.

*“Yes, the working conditions. To start, the climatic conditions make it impossible to do things differently. And then the surgical units are also substandard—you couldn’t say that the unit is systematically sterile.”*

(Operating Room Nurse, Hospital 2)

The capacity of the hospitals to provide the recommended antibiotics plays an important role in the quality of antibiotic prophylaxis practices. Limited financial and logistical resources induced variable and/or incomplete composition of the CS kit in some hospitals. An interviewee mentioned that the antibiotic provided in the kit is the cheapest, and this choice was made for the form.

*“But that’s what’s cheaper, that’s why they put that in the kit to free themselves.”*

(Nurse Anesthetist 2, Hospital 3)

Others suggested that more resources have to be provided to hospitals, since limited resources can lead to an improper antibiotic in the kit, in turn, resulting in improper practice.

*“We must also think about providing public hospitals with resources. Maybe it’s because they don’t have the financial means that they do pretty much.”*

(Nurse Anesthetist 3, Hospital 2)

Regardless of the knowledge of the HcPs on antibiotic prophylaxis or their point of view on the kit, they tended to use what they had available. From the analysis of our data, we understood that, if the contents of the kit provided by the hospital are in line with the recommendations, they will respect the guidance, despite a lack of knowledge or a conflicting point of view on antibiotic prophylaxis. If the antibiotics become available too late, the timing of prophylaxis will not be respected, even when HcPs’ knowledge of correct timing is good.

*“Yes, actually, at a given moment, you use what you have, and that’s it. If you see some Ciplox^®^ (ciprofloxacin) in the box, you use it; if at another time, there isn’t any, you don’t use anything at all.”*

(Anesthetist Medical Doctor 2, Hospital 1)

Disagreement between specialists, preference for departmental habits over central recommendations, and inactive stewardship policies have led to a lack of consensus in the hospitals, which might lead to different practices within the same hospital.

*“It’s here, in reanimation, that our doctors said, ‘No more ampicillin here.’ The antibiotic you should use is co-amoxiclav (amoxicillin + clavulanic acid); at least that’s what we use. That’s not a hospital consensus.”*

(Nurse Anesthetist 1, Hospital 1)

Communication issues at several levels were linked to the lack of consensus and, more generally, to the use of improper antibiotic prophylaxis:

With hierarchy: HcPs reported that administrative managers did not share the CS kit information with all parties concerned, or they shared it with a delay, so the practices in the hospitals did not conform to central recommendations.

*“But apparently it was the hospital managers who have left to some workshops; but they didn’t give us any feedback.”*

(Gynecologist Medical Doctor 1, Hospital 2)

Between specialists: Anesthetists and obstetricians working in the same department had different ideas on antibiotic prophylaxis, and did not make decisions or discuss them together to produce a consensual rule.

*“The gynecologists hold staff meetings every day, and we are invited to the Monday staff meeting. I think the communication methods need to be reviewed because when we say, ‘That’s what needs to be done,’ they come along and do things their way.”*

(Anesthetist Medical Doctor 1, Hospital 1)

Between colleagues: Practitioners in the same field and in the same hospital had divergent opinions on practices of antibiotic prophylaxis for CSs.

*“That’s why I don’t agree completely with my colleague, because she’s not aware of what is in the kit and she hasn’t been curious enough to ask.”*

(Gynecologist Medical Doctor 1, Hospital 2)

Across the patient pathway: Taking care of patients is not a global concept. Oral or written information about patients (patient records) are rare or not considered from one department to another. Each department works unilaterally, driving confusion, omissions, and lowering standards in antibiotic prophylaxis practices.

*“The thing is that here we don’t get any more information about the patients. I don’t know whether there are infections afterwards because we don’t know anything about what happens after day one.”*

(Anesthetist Medical Doctor 2, Hospital 1)

The interviewees highlighted the low activity of the stewardship policy in their hospitals. They then expressed a veritable need of evidence-based medicine and data to improve their practices. They stated that relevant feedback about the biological tests performed and continuous training on antibiotic use are important to improve antibiotic prophylaxis practices.

*“No, not that I know; but if there is a structure like that in place and it was never obvious to them before that they should look at how antibiotic prophylaxis is carried out on women, there is something wrong.”*

(Anesthetist Medical Doctor 2, Hospital 1)

#### 2.2.3. Healthcare Professionals’ Individual Determinants

The HcPs’ views on the cesarean kit, related to their knowledge about antibiotic prophylaxis and their perception of freedom of practice, influence their practices. The HcPs expressed variable confidence in the kit as far as efficacy is concerned. Knowledge about antibiotic prophylaxis, such as the antimicrobial spectrum required, could help them to understand this.

On the one hand, some healthcare professionals perceived the advantages of having a proper antibiotic prophylaxis policy to save money for patients, avoid antimicrobial resistance, and standardize quality practices.

*“Respecting that protocol, which, in my opinion, is good and it avoids patients having to make unnecessary expenditures, could even lessen resistance.”*

(Gynecologist Medical Doctor 1, Hospital 2)

The HcPs appreciated when the contents of the kit allowed antibiotic prophylaxis to be practiced in line with their “*school of thought*” concept.

*“We do what we have seen others do. You cannot just decide to use a third-generation cephalosporin, for example. That’s not in line with what is done.”*

(Anesthetist Medical Doctor 4, Hospital 1)

On the other hand, some of the HcPs preferred an alternative protocol (antibiotic choice) for antibiotic prophylaxis. The HcPs in certain hospitals had more confidence in their personal experiences.

*“No, we draw up our own protocol based on what we have experienced in the service.”*

(Gynecologist Medical Doctor 2, Hospital 3)

Despite their preference for alternative protocols, some practitioners resigned themselves to using the kit.

*“But none of those molecules are available here in Benin. So, we are resigned to that.”*

(Anesthetist Medical Doctor 2, Hospital 1)

An interviewee pointed out the overuse of antibiotics in some interventions as follows:

*“Sometimes, we exaggerate in the antibiotic prophylaxis here. Me, I often have trouble when we want to do clean intervention (for example the planned hernia intervention), and we put Ceftriaxone for prophylaxis.”*

(Nurse Anesthetist 2, Hospital 3)

Leadership in antibiotic prophylaxis decisions also had an impact on the practices. Some of the HcPs considered the kit as a landmark.

*“Antibiotic prophylaxis is based on what’s in the kit. We use the antibiotics that are available in the kit.”*

(Gynecologist Medical Doctor 1, Hospital 2)

Other interviewees stated that the doctor’s decision is the landmark, and that the other members of the team should abide by it. He or she is free to change what they want to use in their practice, and the doctor is not under any influence.

*“In the end, the surgeon (doctor) decides on the antibiotic, which we continue after the surgical unit until the time comes to stop and replace it with oral administration.”*

(Operating Room Nurse, Hospital 2)

*“After the intervention, it is the gynecologist who defines whether we should continue the prophylaxis or not and marks it in the post-operative protocol.”*

(Nurse Anesthetist 2, Hospital 3)

Some of the HcPs feel obliged to follow the kit protocol despite their own opinion, whereas others continue to consider what they were taught in their training.

*“Except that the ampicillin, we use there, I don’t really agree with that. Even if we can go to ceftriaxone, it will be good”*

(Nurse Anesthetist 3, Hospital 3)

*“Because what I was taught about antibiotic prophylaxis when I was in training is that you have to start low and then go up”*

(Nurse Anesthetist 2, Hospital 3)

#### 2.2.4. Central Organizational and Structural Determinants (Policy Management)

The organization and structure of the CS fee exemption policy in Benin were a matter of controversy. Some disagreements on central policy management were reported in all of the hospitals included in our study. In addition to the perceived inefficacy of the kit, the negative point of view of the HcPs on the national policy might influence the implementation of standardized antibiotic prophylaxis.

*“Their kit is useless; when it comes to antibiotic prophylaxis, it’s worthless.”*

(Anesthetist Medical Doctor 3, Hospital 1)

A lack of communication or consensus between policymakers and practitioners emerged in the verbatim transcripts. The practitioners felt uninvolved in the policy formulation and were hesitant.

*“In my opinion, there was no consultation of local healthcare workers about this business of fees exemption in cesarean. It’s a political matter. We were very hesitant at first because the basis for it is not clear.”*

(Anesthetist Medical Doctor 1, Hospital 1)

Low approval of the antibiotics selected in the kit by the central authority, and the absence of monitoring of the utilization of the kit were expressed by the interviewees.

*“We have available to us a prepacked kit that includes an antibiotic that is not indicated for prophylaxis.”*

(Anesthetist Medical Doctor 2, Hospital 1)

#### 2.2.5. Patient Behavior Determinants

The interviewees commonly described patient behavior as a central determinant of the poor achievement of antibiotic prophylaxis practice. The HcPs bemoaned the fact that patients do not bring any antibiotics, do not buy the prescribed quantity of antibiotics, or do not bring them on time. Limited resources for patients (lack of money) to buy antibiotics (some or all doses) was frequently mentioned. Patients also frequently misunderstood the retroactive reimbursement for the CS procedure, and, therefore, refused the additional fees for the antibiotic prescription when CSs are supposed to be exempt of user fees. Thus, we can hypothesize that patients’ behaviors can influence all of the conventional criteria for antibiotic prophylaxis practices.

*“Let me tell you that for some time now, most can no longer manage to pay. They say, ‘we can’t buy it,” and we say, ‘go and buy whatever you can.’ Sometimes some of them come back with two bottles, others with one bottle.”*

(Nurse Anesthetist 1, Hospital 1)

## 3. Discussion

Our study assessed the level of knowledge and explored the beliefs of healthcare professionals on antibiotic prophylaxis in CSs in the context of the user fees exemption policy in three hospitals in Benin. The rate of response of the participants to the survey of knowledge according to the five conventional criteria of antibiotic prophylaxis was approximately 94.3%, which is higher than the 84.8% reported in a Saudi study in 2013 [25], and the 54.7% reported in a Burkinabe study in 2013 [26]. Analysis of the data showed poor scores regarding the knowledge of antibiotic choice, dose administered, and duration of administration. Even if the mean level of knowledge was 3.3 out of 5, we noticed that these three criteria were poorly known and were in line with the results we obtained in a previous observational study [16]. The qualitative data provided some insights to understand the poor practices.

First of all, concerning the choice of antibiotic, the HcPs expressed their disagreement according to the antibiotics selected in the kit, and the CS fee exemption policy in general. Some physicians did not consider the kit safe enough; thus, they were more confident in their own abilities, and preferred to use broad-spectrum antibiotics (contrary to the central recommendation) to avoid the impact of antimicrobial resistance and wound contamination in their patients. In this way, such practices can lead to a vicious cycle of antimicrobial consumption and resistance, as described in a Hungarian study [27]. Moreover, Baadani et al. reported that confidence in one’s prescribing abilities while not recognizing the importance of the guidelines suggests that some physicians may be oblivious to their shortcomings [25]. Echoing our data, 69.1% of respondents in a knowledge survey performed in Burkina Faso declared the use of third-generation cephalosporin in prophylaxis [26]. However, the kit imposes a framework of practices, and some of the HcPs obeyed this, despite their thoughts. Nevertheless, the other HcPs were pleased with the use of the kit, and perceived the use of its antibiotics as a way to improve patient safety and control costs. Moreover, the use of the antibiotic prophylaxis kit resulted in improved conformity of practices with recommendations [21]. The use of a standardized kit supported by strong statements, the hospitals’ management, and the antibiotic stewardship team could help to fix the lack of communication, and provide a consensus within hospitals. The HcPs were looking for some monitoring of infections and evidence-based practices. In some cases, the HcPs adopted a pragmatic, if not resigned, point of view on antibiotic prophylaxis, and used what was available (in the kit or brought by the patients), regardless of their knowledge or preference. Indeed, compliance with antibiotic prophylaxis guidelines depends not only on the physician’s prescription, but also on the availability of the antibiotics in the hospitals, as well as on what the patient can buy and bring in time. In many cases, the patient is expected to buy their antibiotics, but this instruction is not completed due to various reasons.

Second, according to the statements of the interviewees, an improper dose of antibiotic prophylaxis is due to the patients’ behaviors in terms of refusal to buy antibiotics or buying incomplete doses. Curiously, the HcPs’ level of knowledge was also low for this criterion. Thus, we think that the HcPs were less aware of good practices of antibiotic prophylaxis; otherwise, they could coach the patients to provide the correct dose of antibiotics for prophylaxis. Therefore, we can hypothesize that there is a relationship between the level of knowledge and practices. However, in another study, a dissonance was found wherein participants could correctly identify the appropriate use of antibiotics, and yet fail to apply them in practice [28]. Adherence to guidelines for antibiotic prophylaxis remains a challenge, since a previous systematic review observed a significant variation in the outcomes of all of the antibiotic prophylaxis criteria [29].

Third, improper duration can be explained by the fact that the HcPs fear the occurrence of infection after a CS because of the inadequate hospital environment, the patient’s hygiene, and the lack of an evidence-based protocol. In a study performed in Thailand in 2003, an obstetrician argued that post-operative infections affect his reputation, so he tends to overuse antibiotic prophylaxis [30]. In fact, patient hygiene was reported to be an important factor that leads to improper use of antibiotics, especially in low-income countries such as Benin, as women have a suboptimal health status, including a lack of hygiene [22].

In sum, from the analysis of the five determinants, we understand that improper practices can be explained by various reasons at different levels, such as a poor level of knowledge among healthcare professionals on antibiotic prophylaxis and a lack of confidence in the antibiotics in the kit, or the unavailability of infrastructure and financial resources for the adequate achievement of antibiotic prophylaxis and the non-conforming implementation of the policy at the hospital level. At the central level, we found that poor management of the policy, in terms of a lack of communication with local HcPs to ensure their adherence to the policy and to consider their opinions on the choice of antibiotics, contributed to the dysfunctions. It results that the failure of the organization of the policy at these three levels often shifts the responsibility of adequate antibiotic prophylaxis to the patient (or their parents). Even if patients are important members of the healthcare team and participate more in healthcare decision-making [31], they are not skilled in healthcare and cannot understand and weigh the importance of prophylaxis. It becomes essential for policymakers and healthcare professionals to adequately play their roles to ensure patient safety.

This mixed-methods study is the first to assess the level of knowledge and beliefs on antibiotic prophylaxis in CSs in Benin. The survey, combined with qualitative data, helped to understand the link between knowledge and beliefs on the one hand, and their effects on antibiotic prophylaxis practices on the other hand.

This study has some limitations. First, the sample of the survey was small. This was due to the challenge we faced to enroll participants in the study, mostly because we opted to administer the question only on the day we reached the hospital, and to collect the filled questionnaires within a short deadline. Second, all of the included healthcare professionals were from hospitals located in the southern part of the country; thus, they are not entirely representative of the whole country. It will be interesting to perform a large study including other hospitals and more healthcare professionals. Third, in the qualitative study, we did not reach saturation, because some HcPs did not accept being interviewed. However, we think our findings could contribute to improvements, since they have helped to understand that the poor achievement of antibiotic prophylaxis practices according to the five conventional criteria is due to the low level of knowledge and poor management of the policy.

## 4. Materials and Methods

This mixed-methods study encompassed quantitative and qualitative data to assess the level of knowledge and explore beliefs on antibiotic prophylaxis practices in hospitals. After a survey, some interviews were conducted with healthcare professionals. From August to October 2017, through a convergent parallel design study [32], we collected data on the knowledge and beliefs on antibiotic prophylaxis of HcPs in three representative hospitals chosen based on the three levels of the healthcare system in Benin (one national teaching hospital: hosp1; one zonal teaching hospital: hosp2; one confessional private hospital: hosp3). Moreover, in our previous study, we noticed that antibiotic practices are different between the three hospitals [16]. Thus, performing qualitative studies in each kind of hospital can help to determine the challenges faced in these different settings. Quantitative data on the level of knowledge of antibiotics were collected through a survey using a self-administered questionnaire (File S1), whereas qualitative data were gathered through face-to-face interviews and focus groups performed with groups of at least two HcPs with an interview guide (File S2), as described below. All data collection tools were in French, and data collection was conducted in French also.

### 4.1. Assessment of the Level of Knowledge of Antibiotic Prophylaxis

The HcPs who were in the obstetric ward at the time we reached the hospital were informed about the purpose of the study, and the self-administered questionnaire, adapted from those used in a similar study in Burkina Faso [26], was given to those that agreed to participate in the survey (obstetricians, anesthetists, and nurse anesthetists). It comprised 10 multiple-choice questions in order to assess the knowledge of the antibiotic prophylaxis concept and its five conventional criteria (indication, choice of the molecule (ampicillin alone or + gentamicin and/or metronidazole from the kit or cefazoline), dose of antibiotic (double of the usual adult dose), timing (30–60 min before incision), and duration of administration (single administration)) [17].

### 4.2. Interviews

The face-to-face interviews and focus groups were performed in the three hospitals using a semi-structured guide. The HcPs concerned by antibiotic prophylaxis practices in each hospital (anesthetists, specialized anesthetists, nurse anesthetists, and obstetricians) were recruited based on convenience sampling. Depending on the availability of the HcPs, focus groups or face-to-face interviews were organized by physical contact at the sites (wards). The meetings were conducted by the principal investigator of the study (a female external hospital pharmacist and PhD student), who was trained to perform qualitative research. She met the HcPs previously during the observational study conducted in 2016 [16]. The interview guide was open-ended and explored the HcPs’ habits of antibiotic prophylaxis, perceived performance, and issues related to it. The guide was updated after the first interviews, where necessary. After obtaining authorization from the interviewees, the interviews were recorded using the dictaphone of two mobile phones. One of the interviewees did not consent to be recorded, so we then made field notes during the interview.

### 4.3. Data Management and Analysis

Quantitative data were analyzed using IBM Corp (released 2016; IBM SPSS Statistics for Windows, Version 24.0. Armonk, NY, USA: IBM Corp). Descriptive statistics presented continuous variables using medians, and categorical variables were presented as numbers and percentages. The filled questionnaires were scored on a basis of five points considering the five conventional criteria. One point was attributed to each conventional criterion with a good response, and 0 points for a wrong response. The score of each health professional was calculated by summing the points obtained for the five criteria. Each HcP’s knowledge was scored as x/5 (x = total of the points gathered). The mean level of knowledge was calculated by pooling the scores of all participants and dividing the sum by 33. Since the five conventional criteria are essential for the quality of antibiotic prophylaxis, we applied the “all-or-none” law to categorize the level of knowledge of the HcPs. Thus, the level was considered as good when the score was 5/5. Any other score below 5/5 was considered as poor.

In addition, we asked an open question about practice improvement and demographic characteristics (age, gender, qualification, and professional seniority). The completed questionnaires were picked up on the day of the survey or a few days later, according to the HcPs’ availability. All of the collected data were anonymously registered.

All of the collected data were transcribed verbatim in Word software. Each file was named using the words “interview or focus group + the name of the hospital.”

Data analysis was performed by two pharmacists from different countries, including the main investigator. Both were aware of the Benin context, and the main investigator was from Benin. The analysis was performed using inductive content analysis methods—specifically, a “manifest analysis,” in which we stood on “what the interviewees have been said” without straying from our research questions. The analysis comprised approximately five steps, described following a process drawn by Bengtsson [33]. One additional step was used in our case (Decontextualization 2). The steps are summarized in Table S1 in Supplementary Materials. The validity of the analysis was based on the triangulation of information between the survey and the interviews, and the coding process which helped to identify the similarities and differences in the interviewees’ perceptions. Rigor and quality assurance of our study are described in Table S2.

## 5. Conclusions

Cesarean sections can be a life-saving intervention, and antibiotic prophylaxis is an uncontestable parameter to ensure the quality of life and health of patients. Unfortunately, its criteria are insufficiently known by the HcPs in Benin. The lack of confidence in the kit’s antibiotics, the absence of consensus between healthcare professionals, and their disagreement with the national policy were noticed in the hospitals included in our study. This results in failure in the implementation of the policy, and imposes additional fees for patients, despite the user fee exemption for cesarean sections. The healthcare professionals recognized that their practices were not optimal, and attested that they required an operational antibiotics stewardship policy in their hospitals in order to improve said practices. Several determinants involving all actors, implemented on all levels of the healthcare system in Benin, could help to address the challenges of good practices in order to decrease the occurrence of antimicrobial resistance and ensure women’s safety.

## Figures and Tables

**Table 1:** Demographic characteristics of HcPs included in the survey.

Number of HcPs, N	33
Hosp1	29
Hosp2	04
Hosp3	09
Male	17
Female	16
Anesthetists MD	03
Obstetricians	04
Specialized anesthetists	06
Nurse anesthetists	20
Median age (years old)	39.6
(Min: 23–Max: 57)	
Median professional seniority (years)	7.8
(Min: 1–Max: 22)	

**Table 2:** Repartition of the scores of three criteria less-known according to socio-professional categories.

Criteria		Medical Doctor n = 13 (%)	Nurse n = 20 (%)
**Choice of antibiotic**	Ampicillin	2 (15.4)	5 (25.0)
	Amoxicillin + clavulanic acid	6 (46.2)	6 (30.0)
	Cefazoline	3 (23.1)	0 (0.0)
	Ceftriaxone	2 (15.4)	1 (5.0)
	Did not know	0 (0.0)	8 (40.0)
**Dose**	Good	9 (69.2)	8 (40.0)
	Wrong	4 (30.8)	10 (50.0)
**Duration**	Good	10 (76.9)	6 (30.0)
	Wrong	3 (2.3)	14 (70.0)

## Data Availability

Not applicable.

## Supplementary Materials

The following supporting information can be downloaded at: https://www.mdpi.com/article/10.3390/antibiotics11070872/s1. Table S1. Steps of data analysis. Table S2. Rigor and quality assurance. File S1. Questionnaire of assessment of the level of healthcare professionals. File S2. Semi-structured interview guide.
