# Supplementary material for: Healthcare Professionals’ Knowledge and Beliefs on Antibiotic Prophylaxis in Cesarean Section: A Mixed-Methods Study in Benin
Source: Antibiotics (Basel). 2022 Jun 28;11(7):872. doi: 10.3390/antibiotics11070872 (PMC9312278; doi:10.3390/antibiotics11070872)
Supplement: Supplementary file 1 [file antibiotics-11-00872-s001.zip › antibiotics-1742209-supplementary.pdf]

Supplementary Materials:

Table S1. Steps of data analysis

Steps	Activities	How it was conducted
Decontextualization 1	Identification of meaning units	Separate coding was performed by the 2 analysts using the verbatims of 1 face to face interview and 1 focus group
Contextualization	Checking the aspects of the content has been covered regarding the aim of the study	Polling of the codings and live discussion between the 2 analysts.
Categorization	Creation of the categories and themes (codes)	Group the meaning units (codes) into categories and draw the coding tree and validation
Decontextualization 2	Analysis of the remaining interviews based on the coding tree	Coding by the main investigator using the coding tree
Compilation	Creation of the determinants	Performed by the 2 analysts by linking the categories and themes to each "actor" identified in the interviews (e.g.: patients, colleagues, hospital/managers, policymakers)

Table S2. Rigor and quality assurance

**Reflexivity**	• The study is a part of a project which aims to implement a multidisciplinary strategy for prevention and infection control in hospitals. It had a background and focused on a specific aspect of the purpose (antibiotics used in the context of fees exemption policy in cesarean sections), and was designed by pharmacists to understand the healthcare professionals' perceptions of antibiotic prophylaxis.<br>• The main investigator (DAM) has a background in hospital pharmacy and performed an observational study in the obstetric operating rooms. She was trained and had experience in qualitative research methods.<br>• The main investigator and co-authors were not included in the healthcare or the management of the policy in the hospitals to limit the risk of response bias.
**Credibility**	• Participants in the study were involved in the healthcare provided in the hospitals. Data collection was done by the main investigator who designed the study.<br>• Data analysis was performed by 2 pharmacists from different countries including the main investigator. Both are aware of the Benin context.<br>• Data analysis was in five steps (Decontextualization 1: separate codings, Contextualization: pooling and discussions, categorization: drawing the coding tree and its validation, Decontextualization 2: coding based on the coding tree, Compilation: Linking the categories and themes).<br>• Findings were checked with those reported in the literature (worldwide).
**Transferability**	• The qualification and occupation of the participants were variable.<br>• The participants were stakeholders in the healthcare system, and in the management of the obstetric wards in different kinds of hospitals (1st to 3rd levels of the healthcare system).

File S1. Questionnaire of assessment of the level of healthcare professionals.

Antibiotic prophylaxis practices in cesarean section in Benin

Questionnaire for assessment of knowledge level

Hospital /Num : ……………/…….

I- Principles and practice of antibiotic prophylaxis

1- What is the role of antibiotic prophylaxis in surgery?
□ Prevent infection
□ Cure infection
□ Other: ………………………

2- Is antibiotic prophylaxis indicated for cesarean section?
□ Yes □ No

3- Which antibiotics are recommended before cesarean section? …………………………………………………………………………….…………
………………………………………………………………………………………………
………………………………………………………………………………………………
………………………………..

4- Which spectrum of antibiotics is recommended using in prophylaxis?
□ Narrow spectrum
□ Broad spectrum
□ Depends on locale ecology
□ Other: …………………….

5- What is the recommended dose of administration?
□ Usual adult dose.
□ Double of the usual adult dose.
□ Depends on the prescriber.
□ Other: ………………………

6- What is the timing of antibiotic prophylaxis practice?
Time: ……………. minutes
□ Before intervention
□ During intervention
□ After intervention
□ Other: ………………………

7- What is the duration of antibiotic prophylaxis practices?

□ Only one administration
□ 1 to 2 days
□ 3 to 4 days
□ More than 4 days
□ Other: …………………….

8- Is there an antibiotic prophylaxis protocol in your hospital?
□ Yes □ No

9- Is antibiotic prophylaxis practice in your hospital met conventional criteria?
□ Yes □ No

10- Are there improvements to achieve for antibiotic prophylaxis practices in your hospital?
□
Yes ……………………………………………………………………………………………………
……………..……………………………………………….
…………………………………………………………………………………………………
……………………………………………………………………

□ No

II- Demographic characteristics and occupation
Gender: □ Male □ Female
Age: ……… years Seniority: ………… years
Occupation:

□ Anesthetist	□ In specialization	□ Anesthetists nurse
□ Surgeon	□ General practitioner	□ Surgeon assistant
□ Gynecologist	□ Student	□ Nurse/Midwife

File S2. Semi-structured interview guide.

Antibiotic prophylaxis practices in cesarean section in Benin

Semi-structured interview guide

Research question: What are the healthcare workers' beliefs on antibiotic prophylaxis in cesarean section in Benin?

Investigator

I'm DOHOU Angèle, a Hospital Pharmacist and PhD student in clinical Pharmacy. I'm performing my research in four hospitals on the use of antibiotics in cesarean section. I would like to know the point of view of healthcare workers on the use of antibiotics in cesarean sections in Benin.

This interview is lasting approximately 30-45 minutes. I'm going to record it if you allow me. I can give you a transcript of the interview. I ensure you that all information will be used anonymously. There is no wrong response. I'm looking for your personal experience or opinion on the subject.

Please, could you introduce yourself? Give your occupation in the hospital?

Antibiotic prophylaxis practices

1- How antibiotic prophylaxis in the cesarean section is practiced in your hospital. Do practices follow conventional criteria (indication, molecule, dose, timing, and duration)?

2- Why practices are running like that in your hospital?

3- What is your opinion about such practices?

4- How are antibiotics for cesarean are chosen in your hospital? Existence of a special structure working for it?

5- How antibiotics are prescribed to patients for cesarean section? Prescription by a specific person? Existence of an antibiotic prophylaxis protocol? Adherence to protocol? Antibiotics in a cesarean kit are used?

6- Are antibiotics used before cesarean section efficient? How do you assess it? Patient well-being? No infection after cesarean?

7- How do you have information on antibiotic use? Antibiotic prophylaxis? Where do you find them? Professional training? Ongoing training in the hospital? Personal training?
